# Effect of diaphragm and abdominal muscle training on pelvic floor strength and endurance: results of a prospective randomized trial

**DOI:** 10.1038/s41598-019-55724-4

**Published:** 2019-12-16

**Authors:** B. Zachovajeviene, L. Siupsinskas, P. Zachovajevas, Z. Venclovas, D. Milonas

**Affiliations:** 1 Lithuanian University of Health Sciences, Medical Academy, Clinic of Sport Medicine, Kaunas, Lithuania; 2 Lithuanian Sport University, Department of Health Promotion and Rehabilitation, Kaunas, Lithuania; 3 Lithuanian University of Health Sciences, Medical Academy, Department of Urology, Kaunas, Lithuania

**Keywords:** Medical research, Urinary incontinence

## Abstract

Pelvic floor muscles (PFMs) play a crucial role in urinary continence. Therefore, training the PFMs remains the most popular conservative treatment for urinary incontinence (UI). The effect of training other body muscles on the PFMs is unclear and mostly hypothetical. The objective of our study was to evaluate the effectiveness of postoperative diaphragm muscle, abdominal muscle and PFM training on PFM strength (PFMS) and endurance (PFME) as well as on UI in men after radical prostatectomy (RP). Per-protocol PFMS, PFME and urine loss measurements were performed at 1, 3, and 6 months postoperatively. The primary endpoints were PFMS and PFME differences among the study groups. The secondary endpoint was the correlation between UI and PFMS and PFME. In total, 148 men were randomized to the treatment groups. An increase in PFMS and PFME was observed in all groups compared to baseline (p < 0.001). The greatest difference in PFMS was in the PFM training group, but diaphragm training had the best effect on PFME. The highest (from moderate to strong) correlation between UI and PFME and PFMS (r = −0.61 and r = −0.89, respectively) was observed in the diaphragm training group. Despite different but significant effects on PFMS and PFME, all rehabilitation-training programmes decreased UI in men after RP.

## Introduction

Urinary incontinence (UI) is the second most common side effect after radical prostatectomy (RP)^1,2^ and substantially affects a patient’s quality of life. A decade ago, the International Consultation on Incontinence approved training of the pelvic floor muscles (PFM) as an effective treatment option for UI after RP^3^. However, the latest Cochrane Review reported that the value of postoperative PFM exercise following RP remains conflicting^4^, and the recent European Association of Urology (EAU) Guidelines recommended PFM training to speed up recovery from UI^5^. Despite existing controversies on its effectiveness, PFM training is still commonly used for the prevention of UI. At least several important reasons should be mentioned why existing data in this field are conflicting. The first reason is that clinical practice shows that it is difficult to teach men to activate the PFMs properly and to ensure the effectiveness of PFM training even in randomized clinical trials. The second reason is that the intensity of training programmes varies among studies, and this effects the final results regarding incontinence. Finally, there are very little objective data about changes in PFM strength and endurance during the training process and their correlation with UI. Such controversies between real clinical practice and existing study data raise the need for different views on this topic.

During the last two decades, the connection between PFMs and abdominal muscles or the diaphragm has been detected: the PFMs contract and relax during inhalation and exhalation in pace with the diaphragm^6^; the activity of the PFMs increase with the increase of intra-abdominal pressure during forced exhalations or cough^7^. The role of the pelvic floor is essential for the synergy of the diaphragm and abdominal muscles in the maintenance of intra-abdominal pressure^8^. According to the aforementioned physiological functional relationships, we have postulated that diaphragm muscle training (DMT) and abdominal muscle training (AMT) could have similar effects to direct PFM training (PFMT) on pelvic floor muscle strength (PFMS) and pelvic floor muscle endurance (PFME), measured using a perineometer. If our hypothesis is correct, the weaknesses of the existing data could be covered: to teach and control the intensity of diaphragm and abdominal muscle training is easier than PFM training, which ensures quality control; and measuring muscle strength and endurance before and after RP will provide objective data on how different training programmes affect the PFMs and correlate with UI.

Here, we have presented the results of a randomized prospective clinical trial comparing the effect of three different rehabilitation programmes (DMT vs. AMT vs. PFMT) on PFMS, PFME and UI in men after RP resulting from clinically localized prostate cancer (PCa).

## Results

**Figure 1:**
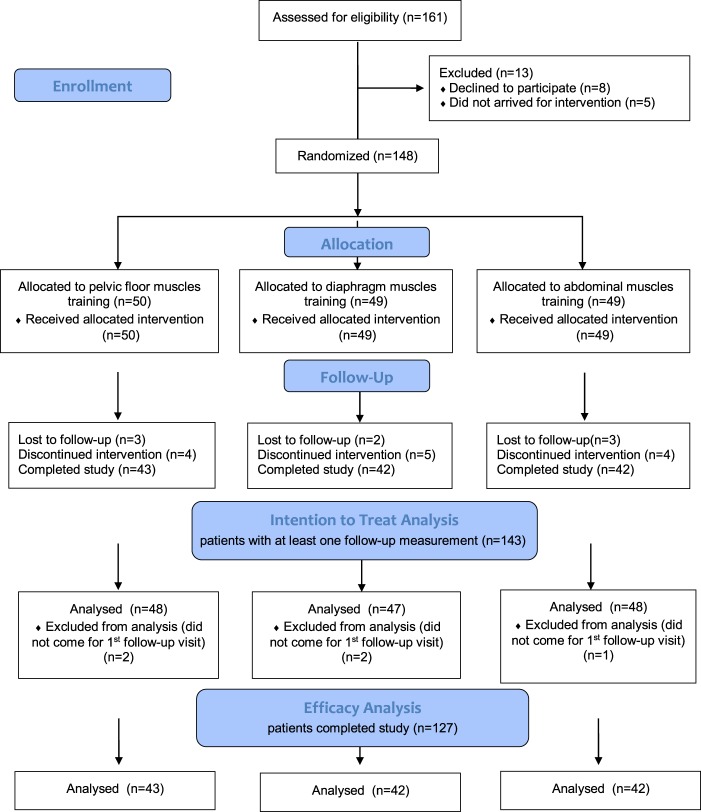
Flowchart of the study.

A total of 148 Caucasian men were randomized to treatment groups between September 2010 and May 2012. A total of 127 men completed the study: 43 in the PFMT, 42 in the DMT and 42 in the AMT groups. The overall dropout rate was 21/148 (14.2%). The reasons for withdrawal from the study were loss of follow-up (n = 8), salvage radiation therapy (n = 4), transportation problems to reach the study centre (n = 3) and discontinuation without explanation (n = 6). The study flowchart is shown in Fig. 1. No additional medical treatment was given because of UI. No adverse events or side effects associated with the training programmes were reported.

### ITT analysis

The ITT population consisted of 143 of the 148 (96.6%) randomized men for whom at least one follow-up visit was performed: 48 in the PFMT and AMT groups and 47 in the DMT group. The baseline patient characteristics that did not differ between groups are shown in Table 1.

**Table 1:** Comparison of the basic clinical and pathological characteristics of the patients (intention-to-treat population) in the study subgroups.

Parameter	PFMT (n = 48)	AMT (n = 47)	DMT (n = 48)	P
Age (year) [mean (SD)]	63.6 (6.1)	64.4 (5.1)	64.3 (4.8)	0.13
PSA (ng/ml) [mean (SD)]	6.8 (4.3)	7.1 (3.3)	6.3 (3.1)	0.53
Prostate volume (ml) [mean (SD)]	40.8 (14.4)	40.6 (13.6)	41.3 (14.1)	0.71
Body mass index [mean (SD)]	28.0 (4.2)	28.1 (3.9)	28.3 (4.0)	0.84
**Pathological stage [n (%)]**				
pT2 pT3a pT3b	30 (62.5) 14 (29.2) 4 (8.3)	30 (63.8) 15 (32) 2 (4.2)	31 (66) 16 (34) —	0.28
**Pathological GS [n (%)]**				
6 7 8–10	9 (18.8) 35 (72.9) 4 (8.3)	10 (21.3) 35 (74.5) 2 (4.2)	11 (23.4) 35 (74.5) 1 (2.1)	0.13
Baseline PFMS [mean (SD)]	91.5 (13.8)	92.1 (10.5)	90.8 (13.1)	0.87
Baseline PFME [mean (SD)]	8.0 (2.7)	7.9 (2.4)	7.9 (2.7)	0.88
UI after catheter removal (g) [mean (SD)]	308.4 (114.6)	303 (91.9)	296.2 (115.2)	0.83

*PFMT* pelvic floor muscle training group, *AMT* abdominal muscle training group, *DMT* diaphragm muscle training group, *SD* standard deviation, *PSA* prostate-specific antigen, *GS* Gleason score, *PFMS* pelvic floor muscles strength, *PFME* pelvic floor muscles endurance, *UI* urinary incontinence.

**Figure 2:**
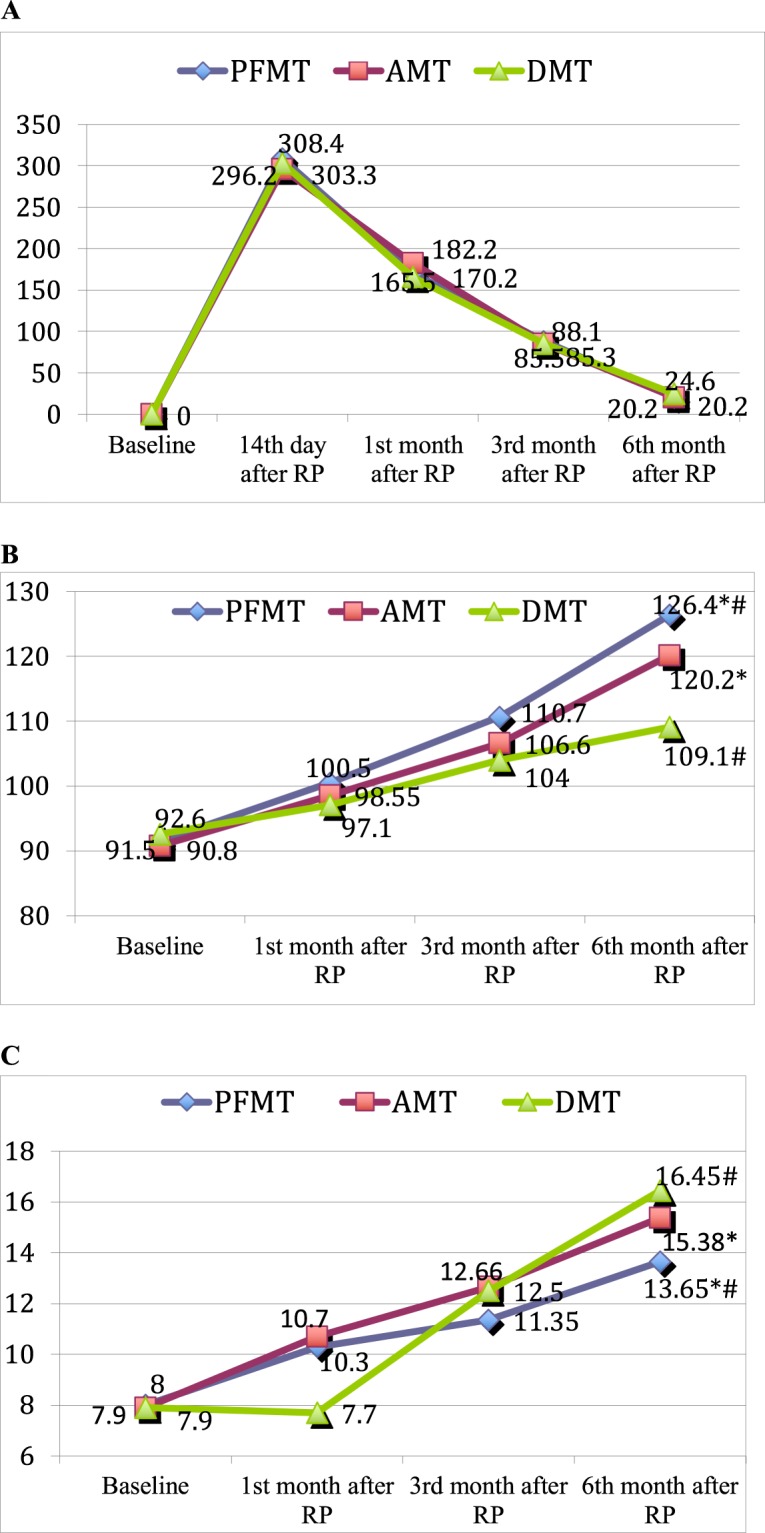
(**A**) Comparison of the changes in urinary incontinence during the 6 months following surgery in the different rehabilitation programme groups. *PFMT* pelvic floor muscle training group, *AMT* abdominal muscle training group, *DMT* diaphragm muscle training group, *RP* radical prostatectomy. (**B**) Comparison of the changes in pelvic floor muscle strength during the 6 months following surgery in the different rehabilitation programme groups. *PFMT* pelvic floor muscle training group, *AMT* abdominal muscle training group, *DMT* diaphragm muscle training group, *RP* radical prostatectomy, * ^#^p < 0.05. (**C**) Comparison of the changes in pelvic floor muscle endurance during the 6 months following surgery in the different rehabilitation programme groups. *PFMT* pelvic floor muscle training group, *AMT* abdominal muscle training group, *DMT* diaphragm muscle training group, *RP* radical prostatectomy, * ^#^p < 0.05.

During the study period, PFMS and PFME increased in all training groups in comparison with those at baseline (all p > 0.001). At the last measurement, PFMS was significantly higher in the PFMT group in comparison with the DMT group (123.6 (95% CI 119.0–128.3) vs. 107.9 (95% CI 104.8–111.1) cmH_2_O, respectively, p < 0.0001) but not with the AMT group (123.6 (95% CI 119.0–128.3) vs. 118.2 (95% CI 114.3–121.9) cmH_2_O, respectively, p = 0.07) (Fig. 2b); PFME was highest in the DMT group (15.8 s, 95% CI 14.7–16.8) which differed in comparison with the PFMT group (13.5 s, 95% CI 12.8–14.1, p < 0.001) but not with the AMT group (15.1 s, 95% CI 14.1–16.0, p = 0.33) (Fig. 2c).

The UI level decreased significantly during each follow-up visit in all groups compared to those at baseline: all p < 0.001 (Fig. 2a). At the last measurement, the UI values were 32.9 (95% CI 17.9–47.9), 38.9 (95% CI 22.6–55.3) and 31.7 (95% CI 19.5–43.9) gr. for the PFMT, DMT and AMT groups, respectively, with no difference between the groups (ANOVA p > 0.75).

Weak to strong reverse correlations were determined between the PFME, PFMS and UI levels at 1, 3 and 6 months postsurgery in all groups (Table 2). The strongest correlations between PFMS and UI and between PFME and UI were detected in the DMT group: r = −0.89, p < 0.0001 and r = −0.61, p < 0.0001, respectively. Increases in PFMS during the study correlated better with UI than did increases in PFME in all groups: r = −0.47 to −0.89 vs. r = −0.54 to −0.61, respectively (Table 2).

**Table 2:** Correlation between pelvic floor muscle strength, endurance and urinary incontinence in the study subgroups.

	Correlation coefficient and Intensity 1 month after surgery			Correlation coefficient and Intensity 3 month after surgery			Correlation coefficient and Intensity 6 month after surgery		
	PFMT group	AMT group	DMT group	PFMT group	AMT group	DMT group	PFMT group	AMT group	DMT group
PFMS - UI	Weak (r = −0.39)	Weak (r = −0.43	Strong (r = −0.85)	Moderate (r = −0.54)	Moderate (r = −0.53)	Strong (r = −0.89)	Moderate (r = −0.68)	Weak (r = −0.47)	Strong (r = −0.89)
PFME - UI	Weak (r = −0.37)	Weak (r = −0.45)	Moderate (r = −0.51)	Weak (r = −0.40)	Weak (r = −0.37)	Moderate (r = −0.61)	Moderate (r = −0.54)	Moderate (r = −0.54)	Moderate (r = −0.61)

*PFMT* pelvic floor muscle training group, *AMT* abdominal muscle training group, *DMT* diaphragm muscle training group, *PFMS* pelvic floor muscle strength, *PFME* pelvic floor muscle endurance, *UI* urinary incontinence.

### Efficacy analysis

This analysis included men who completed the study (n = 127). At the end of the study, PFMS was significantly higher in the PFMT group in comparison with the DMT group (126.4 (95% CI 121.9–130.8) vs. 109.1 (95% CI 105.9–112.4) cmH_2_O, p < 0.0001) and with the AMT group (120.2 (95% CI 116.5–123.8) cmH_2_O, p = 0.03). PFME was the highest in the DMT group (16.4 s, 95% CI 15.4–17.5) and different in comparison with the PFMT group (13.6 s, 95% CI 12.9–14.3, p < 0.0001) but not with the AMT group (15.4 s, 95% CI 14.4–16.4, p = 0.13).

At the end of the study, UI were 20.2 (95% CI 14.4–26.1), 24.6 (95% CI 17.5–31.7) and 20.2 (95% CI 14.5–26.0) gr. for the PFMT, DMT and AMT groups, respectively, with no difference between the groups (ANOVA p > 0.51).

Both analyses confirmed that during the study, PFMS mostly increased in the PFMT group and PFME mostly increased in the DMT group. All training programmes had a similar impact on UI.

## Discussion

Several different risk factors might have influenced UI after RP: preoperative detrusor condition, bladder outlet obstruction, UI before surgery, age, body mass index and some anatomical conditions such as the thickness of the pelvic diaphragm, the functional length of urethra or the prostate volume^3,9–13^; intra-operative techniques such as bilateral neurovascular bundle sparing, bladder neck preservation or urethral suspension^3,14–16^ and postoperative detrusor disfunction, reduced bladder compliance or intrinsic urethral sphincter deficiency^9,17–19^ may also influence UI after RP.

All our patients were continent before RP, and previous prostate surgery was one of the exclusion criteria for the study. There were no differences according to patient age, prostate volume or body mass index between the study subgroups. Bladder outlet obstruction and detrusor overactivity were not specifically investigated in our study, but only a few men had used medications because of lower urinary tract symptoms before RP and no one used them for an overactive bladder. Detrusor instability is common enough condition after RP and is usually treated by anticholinergic medication. Medications for better UI control for an overactive bladder was suspected were not recommended during the study period to avoid misinterpretation of the final results. Urodynamic studies have shown that bladder overactivity, reduced bladder compliance and sphincter insufficiency can be detected in 0–100% of patients with UI after RP; therefore, intrinsic sphincter deficiency represents the main diagnosis in 55% of incontinent patients^19^. The goal of PFM training, also known as Kegel exercises, is to improve the strength and endurance of the striated muscles of the pelvic floor, partially compensating for urethral sphincter insufficiency^4^, and to reduce UI. The present study shows a new approach to PFM activation with a positive effect on PFMS, PFME and UI in men after RP.

A number of scientists agree that there is a relationship between the pelvic floor, diaphragm, low back and abdominal muscles, but the co-activation and inhibition of these muscles in men after RP are still uncharacterized^8,20–23^. Moreover, there is a lack of functional parameters confirming such correlations^8,24–26^, and there is no data regarding men after RP. Our findings revealed that DMT, AMT and PFMT programmes have continuous, significant effects on PFMS and PFME in men after RP. The decrease in UI significantly correlated with increases in both –PFMS and PFME, but the strongest correlation was detected in PFMS. To our knowledge, this is the first report of how diaphragm and abdominal muscle training affect PFMs and UI in men after RP.

The largest change in PFMS compared to baseline was detected in the PFMT group. Differences in PFMS (91.5 vs. 100.5 cm H_2_O, p < 0.01) in this group were observed even after 1 month compared to the preoperative level, and this difference continued to grow during throughout the study (Fig. 2b), which demonstrates the continuous effect of the training. *et al*.ted similar results indicating that PFM training increased the strength of these muscles one year postsurgery^27^, but only one publication presenting the numeric values of PFMS measured with a perineometer was found. The authors found that 6 months after the surgery, the average PFMS value was 64 cmH_2_O, after training the PFMs 2 times per week under the supervision of a physiotherapist^28^. Our data revealed that the mean PFMS 6 months after the RP was 2-fold higher − 126.2 cmH_2_O. Such a difference might be influenced by the frequency of training sessions and the motivation of the patient. More interesting findings in our study were detected in the DMT and AMT groups. The increase of PFMS in these groups was slower and not as high compared to the PFMS in the PFMT group, but PFMS was significantly different at 6 months compared to baseline (93 vs. 109 and 91 vs. 101 cmH_2_O for the DMT and AMT groups, respectively p < 0.001). This clearly shows the presence of functional connections between the abdominal muscles, the diaphragm and the pelvic floor and confirms the conclusion reached by Hodges *et al*. that the activity of the PFMs increases during the inhalation phase when the diaphragm is descending^29^.

The increase of PFME in the DMT group (Fig. 2C) was strongly expressed, and at 6 months, it reached a significant difference compared to the PFME in the PFMT group (16.4 vs. 13.6 s, respectively, p < 0.001) but not to the AMT group (16.4 vs. 15.4 s, respectively, p = 0.13). A decrease in PFME during the first month in the DMT group could be explained by difficulties with tonic muscle activation because of the urethral catheter. After catheter removal, a rapid increase in PFME was registered. Such an unexpected finding could be explained using the hypothesis that intra-abdominal pressure is increasing during inhalation and tonic muscle fibres of the pelvic floor are “trained” for endurance. This idea has been supported by several authors showing that during DMT, m. pubococcygeus (part of the pelvic floor) ensures postural control of the pelvic floor. During DMT, concentric contraction of this muscle causes ascension and descension during the eccentric contraction^26,30,31^. This could be the reason why DMT had a significantly higher impact on PFME compared to PFMT. Despite the fact that in a number of publications, the association between the pelvic floor and the diaphragm has been presented, there is a lack of functional parameters showing that the relationship^8,24–26,30^. Our study is the first to confirm such an association in a cohort of men after RP.

Urine loss decreased throughout the study period (Fig. 2A), and at 6 months, it ranged from 20.2 to 24.2 ml, with no difference among the study groups (ANOVA p < 0.5). For the evaluation of UI, we used the 8-hr pad test. Considering that the sensitivity and specificity of the 1-hr pad test varies significantly in the literature^32^ and prolonged tests are recommended^33^, the 8-hr test could provide objective information about UI during daily activities. For better interpretation of our results, we used the following 1-hr pad test UI description: 0–1 gr/hr – continent; 1–10 gr./hr – mild incontinence; and 10–50 gr/hr – moderate incontinence. A total of 35.4% of the men were absolutely continent (0–5 gr/8 hr or 0–1 gr/1 hr) 6 months after RP, with no difference between the PFMT (37.2%) and the DMT (38.1%) and AMT (31.0%) groups (p = 0.76). Other men had mild incontinence (8–75 gr/8 hr or 1–10 gr/1 hr). Because detailed analysis of the UI rate was not the main goal of the study, for further analysis, we used only the value of urine loss in ml that most precisely reflected the links between muscle function and incontinence.

The exact role of PFMS and PFME in continence is still unknown and should be determined. Regarding our study results, some tendencies could be observed by analysing the correlation between the PFM condition and UI. In general, the correlation between PFMS, PFME and UI increased during each follow-up visit in all subgroups. This suggests that continued rehabilitation programmes should be used for at least 6 months with training directed at PFME and PFMS. The strongest correlation was found in the DMT group at all study visits, which confirms a direct connection between the diaphragm and the pelvis muscles. *et al*.uded that the PFMs play an important role in the physiology of breathing. PFMs not only support pelvic organs but also participate in the dynamic control of intra-abdominal pressure together with inhalation and exhalation^25^. The functional synergy between these muscles is very important for understanding how to provide better control of UI not only in such a cohort of men but possibly also for women with UI.

PFMS had a higher correlation with UI compared to PFME in all study subgroups. One possible explanation for this finding could be that stress incontinence is common at the first months after RP. The sudden increasing of abdominal pressure requires a quick reaction of the muscles. Stronger PFMs might be more effective in resisting sudden increased abdominal pressure in cases of stress UI that indirectly protect internal and external urethral sphincters that enter the m. transversus perinei profundus, which is part of the medial layer of the PFMs. PFME and PFMS have a relationship between each other: fast fibres are more active during quick and strong contractions, and slow fibres are involved in endurance exercises. However, in general, according to our subgroup data, synergistic but not concurrent relations between muscle strength and endurance offer optimal results for UI control.

To summarize our study findings, several important messages should be emphasized. The first is that DMT and AMT significantly, but to different degree than PFMT, influence PFMS and PFME. The second is that DMT and AMT reduce UI to the same degree as PFMT. The third is that the correlation between UI and PFMS dynamics is stronger than that between UI and PFME. The fourth is that despite some controversies in our findings, all training programmes could be designed to effect PFM strength and endurance. Finally, it is difficult for both patients and physicians to control the quality of PFM exercises; therefore, breathing and abdominal wall exercises can be suggested as a very effective alternative for men after RP.

There are several limitations that might impact the interpretation of our study results. The absence of a control group did not allow us to prove the effect of postoperative exercises on PFMS and PFME. A control group should be included in future studies to test the true therapeutic effect of rehabilitation programmes because continence could improve with a time without any muscles training. Involvement in the study without any treatment was not acceptable for our patients and was a reason for the lack of a control group. Formation of a control group will be a challenge for future studies. The six-month duration of the study was another limitation. PFMS and PFME increased throughout the study period and still had not reached a plateau that could influence the real UI rate. The 8-hr pad test allowed us to evaluate UI during the most active time of the day; however, the comparison of our results with the standardized 1-hr test is conflicting. Finally, the objective investigation of lower urinary tract and detrusor instability symptoms before surgery, let exclude one of possible reasons for persisting UI.

The same investigator and strict methodology ensured the quality of performing the PFM and UI measurements, and this is the most important strength of the study. Similar patient baseline characteristics and a randomized prospective study design are two other strengths of our study. To our knowledge, the effectiveness of breathing and abdominal wall exercises on PFMS, PFME and UI after RP are presented here for the first time, and this makes it impossible to compare our results with those of other researchers in the field. More randomized controlled trials are needed to confirm our findings.

## Conclusions

Diaphragm, abdominal wall and pelvic floor muscle training had a different influence on pelvic floor muscle strength and endurance but the same effect on the decrease of urine loss in men after radical prostatectomy. Pelvis muscle strength correlated better with a decrease in urine loss than did muscle endurance.

The highest correlations between pelvic floor muscle strength and urine loss and between pelvic floor muscle endurance and urine loss were in the diaphragm muscle training group. All three training programmes could be suggested for patients if rehabilitation for urinary incontinence after radical prostatectomy is appointed.

## Methods

This was a single-centre, randomized, parallel-group, non-inferiority study conducted between September 2010 and May 2012 at the Lithuanian University of Health Sciences. Participants were randomly assigned (without stratification by patient age or body mass index or other parameters) with an allocation ratio of 1:1:1 to receive diaphragm muscle training (DMT), abdominal muscle training (AMT) or PFM training (PFMT) for 6 months. The intervention groups were assigned randomly in the absence of an investigator. After the investigator evaluated the data and before application of a particular intervention programme, a list of subjects was presented to the rehabilitation institution where the particular intervention was applied. Without viewing the measurement data for the subjects, the institution assigned one of the 3 intervention programmes to each subject using numbered envelopes.

The primary endpoint of this study was to compare the effect of activation of different muscle groups on PFMS and PFME. The secondary endpoints included the correlations between UI and PFMS and PFME dynamics for 6 months postoperatively. The study protocol was approved by the Regional Biomedical Research Ethics Committee (Protocol ID BE-2-61), and the data were processed after the permission of the State Data Protection Inspectorate (Protocol ID 2R-1697). The trial was registered in the ClinicalTrials.gov database (NCT03858452, 27/02/2019). We confirm that all research was performed in accordance with relevant guidelines and regulations. All patients signed a written informed consent form before enrolment in the study.

### Eligibility

Eligible participants were men aged 45 to 75 years who underwent RP because of clinically localized PCa. The inclusion criteria were a stable somatic state, no chronic obstructive pulmonary disease, no surgical interventions in the abdominal area, no complaints in the lower back, no acute musculoskeletal injuries in the last 6 months and no cognitive dysfunction.

### Assessments during the study

Medical history, physical examination, PFMS and PFME were recorded the day before the RP and at 1, 3 and 6 months at per-protocol visits after RP.

PFMS and PFME were measured using the perineometer “Peritron 9300 A” (Cardio Design Pty. Ltd. Australia). The anal sensor of the perineometer was inserted into the anus. To evaluate PFMS, the subjects were asked to maximally contract the muscles of the anus. To evaluate PFME, the subjects were asked to hold the contracted muscles of the anus as long as possible. The endurance result was recorded when the maximal value dropped by more than 5 cm H_2_O. The maximal error of the device is <0.7 cm H_2_O. The measurement was performed once. Patients were educated about the localization and function of PFMs before the measurements and were encouraged to activate the muscles properly during the test.

UI was measured on the day of catheter removal and during the 1-, 3- and 6-month visits using the 8-hr pad test. The first pad test was performed with the catheter removed, usually 14 days after RP. The subject was asked to weigh the pad during the course of the 8 hrs and to record the weight having subtracted the weight of the pad itself. The regularly recommended pad weight was 90 g. In the case of greater UI, the subjects could use a larger pad of 140 g. The smallest pad used by the subjects weighed 40 g. The pads were weighed using electronic scales. The accuracy of the device was ±2 g. On the day of catheter removal, a pad was worn from 10 am to 6 pm (±10 minutes). During repeated pad tests, the subject was asked to wear a pad 8 hrs per day after morning urination on the test day.

Physiotherapy started on days 7–9 following surgery. The patients performed therapeutic exercises under the supervision of a physiotherapist for a total of 16 days. Until the removal of the catheter, personalized physiotherapy sessions were conducted.

In the PFMT group, the exercises were performed in various positions: laying on the back with bent legs and a lifted pelvis, laying on the back with bent legs, sitting on a gym ball, standing, walking and using the stairs. Activation of the PFMs consisted of short dynamic contractions, 2–3 sets in one session with a 1-minute break, gradually increasing the repetitions. During urination, we recommended that patients stop the flow 2 times. PFM contraction was ensured by asking the patient to make the pelvic floor “concave”. The exercises were performed twice per day for approximately 30 minutes.

In the DMT group, the breathing exercises were performed in various positions: while lying on the floor with bent or extended legs, while in a quadruped position, and while performing small squats holding the arms on the thighs. We used strong and hard concentric and eccentric contractions of the diaphragm with resistance. For activation of the diaphragm, subjects were instructed to do the following: inhale through the nose, exhale slowly through the mouth, inhale through one nostril, exhale through the mouth, inhale and exhale through the nose, inhale through the nose, and exhale through the mouth while limiting the chest expansion with a belt. All the exercises were performed as 2 sets of 6–8 repetitions with a 1-minute break twice a day for 30 minutes, gradually increasing the intensity.

In the AMT group, at the beginning, the subjects were taught correct activation of the abdominal muscles: retraction of the abdomen was performed during the expiration while the pelvic and torso area remained stable. Lower limb movements were introduced to progress the training. A concentric muscle contraction regimen was applied. Activation exercises of the transversus abdominis muscle were performed in different positions: with the subject lying on the back with bent and straight legs; lying on the abdomen; on the knees with the hands placed on the ground; standing with the body and shins insignificantly bent and with the arms resting on the thighs; walking; and climbing the stairs. The exercises were performed twice per day for 30 minutes.

### Statistical analysis

In the present study, the necessary sample size was calculated (for each group) based on the outcomes for pelvic floor muscle strength and endurance. The sample size was calculated for each indicator separately, and the higher value was chosen as the necessary sample size. After 10 pilot measurements were taken, it was determined that the average pelvic floor muscle activity and its standard deviation was 89.6 ± 13.3 cm H_2_O and that pelvic floor endurance was 8.0 ± 2.6 s with a 10% permitted inaccuracy of the average value. Sample size calculations showed that 8 patients were needed for each group to compare pelvic floor strength and 40 patients were needed to compare pelvic floor endurance. The latter value, as the higher value, was chosen as the necessary sample size for each of the 3 groups of subjects.

An intention-to-treat (ITT) analysis was performed for patients who underwent at least one per-protocol UI and PFM measurement. An efficacy analysis was performed for patients who completed the study.

The quantitative variables are presented as the mean (m) and standard deviation (SD). Differences between the first and second, second and third, and third and fourth measurements were evaluated by calculating the absolute difference of the averages and the percentage difference. The nonparametric Kruskal-Wallis test was used to evaluate independent variables, and the nonparametric Friedman test was used to evaluate the dependent variables. The frequency of qualitative characteristics was expressed in percent, and their interdependence was evaluated by the chi-squared criterion *(χ*^2^*)*. To evaluate functional relations, the Spearman correlation coefficient r was chosen. If r = 0, there was no correlation between the observed variables. If 0 < |r| ≤ 0.3, the correlation was very weak; if 0.3 < |r| ≤ 0.5, it was weak; if 0.5 < |r| ≤ 0.7, it was medium; if 0.7 < |r| ≤ 0.9, it was strong; and if 0.9 < |r| ≤ 1, it was very strong. To check the statistical hypotheses, the 95% significant difference level was chosen (P < 0.05).
